# Carbapenem susceptibilities of Gram-negative pathogens in intra-abdominal and urinary tract infections: updated report of SMART 2015 in China

**DOI:** 10.1186/s12879-018-3405-1

**Published:** 2018-09-29

**Authors:** Hui Zhang, Haishen Kong, Yunsong Yu, Anhua Wu, Qiong Duan, Xiaofeng Jiang, Shufang Zhang, Ziyong Sun, Yuxing Ni, Weiping Wang, Yong Wang, Kang Liao, Huayin Li, Chunxia Yang, Wenxiang Huang, Bingdong Gui, Bin Shan, Robert Badal, Qiwen Yang, Yingchun Xu

**Affiliations:** 1 Division of Microbiology, Peking Union Medical College Hospital, Peking Union Medical College, Chinese Academy of Medical Sciences, No. 1 Shuaifuyuan, Wangfujing Street, Beijing, 100730 China; 2 Department of Microbiology, The First Affiliated Hospital of Zhejiang University, Hangzhou, 310003 China; 3 Department of Infectious Diseases, SirRunRun Shaw Hospital, School of Medicine, Zhejiang University, Hangzhou, 310016 China; 4 Infection Control Center, Xiangya Hospital, Central South University, Changsha, 410008 China; 5 Microbiology Laboratory, Jilin Province People’s Hospital, Changchun, 130021 China; 6 The Fourth Hospital of Harbin Medical University, No. 37 Yiyuan Road, Nangang District, Harbin, China; 7 Division of Microbiology, Haikou People’s Hospital, Haikou, 570208 China; 8 Department of Laboratory Medicine, Tongji Hospital, Tongji Medical College, Huazhong University of Science and Technology, Wuhan, 430030 China; 9 Division of Microbiology, Ruijin Hospital, School of Medicine, Shanghai Jiaotong University, Shanghai, 200025 China; 10 Nanjing General Hospital, No. 305 Zhongshan Dong Road, Nanjing, China; 11 Department of Laboratory Medicine, Shandong Provincial Hospital Affiliated to Shandong University, Jinan, 250021 China; 12 Division of Microbiology, The First Affiliated Hospital, Sun Yat-Sen University, Guangzhou, 510080 China; 13 Zhongshan Hospital Affiliated to Fudan University, No. 180 Fenglin Road, Shanghai, 200032 China; 14 Beijing Chao-yang Hospital, 8 Gongren Tiyuchang Nanlu, Chaoyang District, Beijing, 100020 China; 15 Division of Microbiology, The First Affiliated Hospital of Chongqing Medical University, Chongqing, 400016 China; 16 Clinical laboratory, The Second Affiliated Hospital of Nanchang University, Nanchang, 330006 China; 17 First Affiliated Hospital of Kunming Medical University, No. 295 Xichang Road, Kunming, 650032 China; 18 Division of Microbiology, International Health Management Associates, Schaumburg, IL 60173-3817 USA

**Keywords:** *Enterobacteriaceae*, Carbapenem; ertapenem, Imipenem, Intra-abdominal infection, Urinary tract infection

## Abstract

**Background:**

To evaluate the susceptibility rates of aerobic and facultative Gram-negative bacterial isolates from Chinese intra-abdominal infections (IAI) and urinary tract infections (UTI) focusing on carbapenems and comparing their effectiveness between 2014 and 2015.

**Methods:**

A total of 2318 strains in 2015 (1483 from IAI and 835 from UTI) and 2374 strains in 2014 (1438 from IAI and 936 from UTI) were included in the analysis. Antimicrobial susceptibilities were determined at a central laboratory using CLSI broth microdilution and interpretive standards. Hospital acquired (HA) IAI and UTI were defined as isolates sampled > 48 h and community acquired (CA) as isolates sampled < 48 h after admission.

**Results:**

The main species derived from IAI and UTI in 2015 were *Escherichia coli* (50.86%) and *Klebsiella pneumoniae* (19.20%). Susceptibilities of *Escherichia coli* IAI and UTI strains to imipenem (IPM) and ertapenem (ETP) were > 90% in 2014 and 2015, while the susceptibilities to IPM and ETP of *Klebsiella pneumoniae* IAI strains were >  80% in 2014 but dropped to ≤80% in 2015 for UTI strains. Susceptibilities of IAI *Enterobacteriaceae* strains to IPM and ETP in 2015 were lowest in the colon and abscesses, and *Enterobacteriaceae* susceptibilities of UTI and IAI isolates to IPM and ETP were lowest in medical, pediatric and surgery intensive care units (ICUs) in 2015.

**Conclusions:**

IPM and ETP were effective in vitro against *Enterobacteriaceae* isolated from IAIs and UTIs in 2014 and 2015, but susceptibility to carbapenems in UTIs markedly decreased in 2015.

**Electronic supplementary material:**

The online version of this article (10.1186/s12879-018-3405-1) contains supplementary material, which is available to authorized users.

## Background

The Study for Monitoring Antimicrobial Resistance Trends (SMART)-CHINA is a surveillance program which monitors annually in vitro activities of antimicrobial agents against pathogens that cause intra-abdominal infections (IAI) and urinary tract infections (UTI). In a previous Chinese study it was reported that the incidence of Extended-Spectrum β-Lactamases (ESBL)-producing *Escherichia coli* (*E. coli*) strains derived from IAI had significantly increased between 2002 and 2011, while the percentages of ESBL-producing *Klebsiella pneumoniae (K. pneumoniae*) strains isolated from IAI remained relatively constant between 30.1 and 39.3%, but these two species were the major pathogens during the entire period [1]. However, Asia has been reported to have the world’s highest incidence of ESBL-producing *E. coli*, *K. pneumoniae*, *Klebsiella oxytoca* and *Proteus mirabilis* strains from IAIs and UTIs in 2011, reaching 40% to 45% [2], and a number of recent publications have noted that cephalosporins and fluoroquinolones were not suitable antibiotics for the empirical treatment of IAI and UTI in China [3, 4], underlining the importance of monitoring susceptibilities to alternative antibiotics such as the carbapenems. Epidemiological and individual hospital drug susceptibilities are commonly used as a guide for selecting suitable antibiotics for empirical treatments. However, susceptibility analyses have been extended to weighted-incidence syndromic combination antibiograms (WISCA), which reflects the likelihood that regimens treat all relevant organisms in a patient with a given syndrome [5].

In the present study we developed organ-specific weighted incidence antibiograms (OSWIAs) to estimate the likelihood of an isolate from a specific organ being susceptible to a given antibiotic. IAI and UTI derived isolates and their susceptibilities to carbapenems, cephalosporins, fluoroquinolones, broad-spectrum penicillins combined with β-lactamase inhibitors, and an aminoglycoside were compared in different infected organs. In addition, the distribution of *Enterobacteriaceae* and non-*Enterobacteriaceae* infections isolated from HA and CA IAIs and UTIs in different age subgroups as well as the susceptibility patterns of major pathogens in different medical departments were also analyzed.

## Methods

### Isolates from IAI and UTI infections

The Human Research Ethics Committee of Peking Union Medical College Hospital approved this study and waived the need for consent (Ethics Approval Number: S-K238). A total of 2318 aerobic and facultative Gram-negative bacterial strains (1483 from IAI and 835 from UTI) in 2015 and 2374 strains in 2014 (1438 from IAI and 936 from UTI) collected from 21 hospitals in 16 Chinese cities were retrospectively analyzed. The majority of the intra-abdominal specimens were obtained during surgery, with some paracentesis specimens. The UTI isolates were obtained from clean catch midstream urine, the urinary bladder, kidney and the prostate gland. All duplicate isolates (the same genus and species from the same patient) were excluded. Bacteria were identified by standard methods used in the participating clinical microbiology laboratories. Isolates were considered to be community-associated (CA) if they were recovered from a specimen taken < 48 h after the patient was admitted to a hospital, or HA if the specimen was taken ≥48 h after admission, as previously described [6].

### Antimicrobial susceptibility test methods

Minimum inhibitory concentrations (MIC) were determined by broth microdilution according to the Clinical and Laboratory Standards Institute (CLSI) [7] using panels purchased from ThermoFisher Scientific (Cleveland, OH, USA). Relative susceptibility interpretations were based on CLSI clinical breakpoints [8].

Twelve antimicrobial agents commonly used to treat IAI and UTI were tested: ampicillin-sulbactam (SAM), piperacillin-tazobactam (TZP), ceftriaxone (CRO), cefotaxime (CTX), ceftazidime (CAZ), cefoxitin (FOX), cefepime (FEP), ciprofloxacin (CIP), levofloxacin (LVX), amikacin (AMK), imipenem (IPM) and ertapenem (ETP). Reference strains *E. coli* ATCC (American Type Culture Collection) 25,922, *Pseudomonas aeruginosa* ATCC 27853, and *K. pneumoniae* ATCC 700603 (positive ESBL control), were used as quality control (QC) strains for each batch of MIC tests. Results were only included in the analysis when corresponding quality control isolate test results were in accordance with CLSI guidelines and therefore within an acceptable range.

### Organ-specific weighted incidence antibiogram (OSWIA) calculation

In order to calculate bacterial sensitivities in various organs of the abdominal cavity OSWIAs were calculated using the following equation:

Weighted susceptibility of a certain antimicrobial drug in a certain organ = antimicrobial susceptibility of A × the constituent ratio of A in the organ + antimicrobial susceptibility of B × the constituent ratio of B in the organ + antimicrobial susceptibility of C × the constituent ratio of C in the organ +… (where A, B and C represent the pathogenic bacteria in a certain organ).

For example when we calculated the OSWIA susceptibility of gall bladder isolates to ETP in 2015, first we extracted specific bacterial infection rates and then multiplied them by the specific bacterial susceptibilities to ETP in 2015. Isolates in 2015 from gall bladder were 241 *Escherichia coli* (50.1%), 87 *Klebsiella pneumoniae* (18.1%), 36 *Enterobacter cloacae* (7.5%)……until 1 *Serratia odorifera* (0.21%). The corresponding susceptibilities to ETP were 87.55% (*Escherichia coli*), 86.21% (*Klebsiella pneumoniae*), 66.67% (*Enterobacter cloacae*)……and 100% (*Serratia odorifera*). According to the above mentioned equation, the susceptibility of gall bladder to ETP was calculated as 50.1% × 87.55% + 18.1% × 86.21% + 7.5% × 66.67%......0.21% × 100% = 85.79%.

### Statistical analysis

The susceptibility of all Gram-negative isolates combined was calculated using breakpoints appropriate for each species and assuming 0% susceptible for species with no breakpoints for any given drug. The 95% confidence intervals (CIs) were calculated using the adjusted Wald method; linear trends of ESBL rates in different years were assessed for statistical significance using the Cochran-Armitage test and comparison of ESBL rates were assessed using a chi-squared test. *P*-values < 0.05 were considered to be statistically significant.

## Results

### Distribution of Enterobacteriaceae isolates acquired from different organ groups of IAI and UTI in 2015

**Fig. 1:**
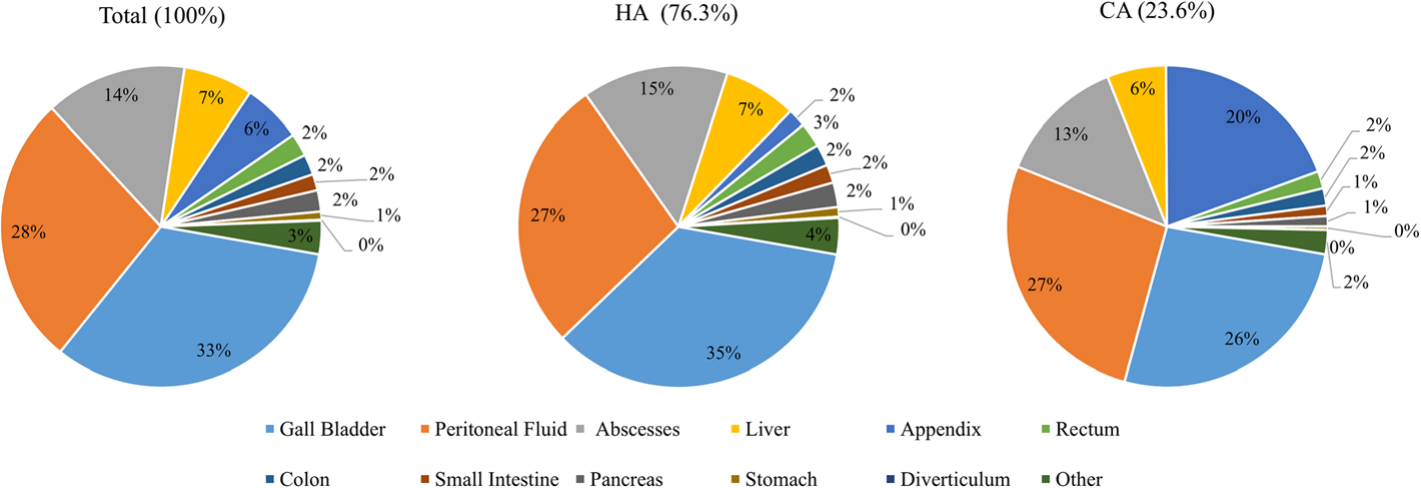
Distribution of isolates acquired from IAI pathogens in different organ groups in 2015

*Enterobacteriaceae* strains of IAIs were mainly derived from gall bladder (33%), peritoneal fluid (28%), abscesses (14%) liver (7%), appendix (6%) and other organs (1–3%). Comparing *Enterobacteriaceae* strains acquired from HA (76.3%) and CA (23.6%) IAI, the percentages for peritoneal fluid, abscesses and liver-derived isolates were similar, but in HA-derived IAIs, gall bladder isolates were collected more often (35% vs 26%), whereas appendix isolates were less frequently sampled (2% vs 20%) compared to CA isolates (Fig. 1). Similar distributions were found in 2014 (Additional file 1: Figure S1). From UTI pathogens, 99.4% of the strains were acquired from urine, with only 3 strains from the kidneys, and 1 strain each from the bladder and prostate gland. The distribution of HA and CA UTI isolates was 599 (71.9%) and 234 (28.1%) in 2015, and 58.1% and 40.9% in 2014, respectively.

### Distribution of all isolates acquired from HA and CA IAI and UTI in 2015

There were 2318 strains collected in 2015, including 1483 strains from IAIs and 835 strains from UTIs. The majority of infections were caused by *E. coli* and *K. pneumoniae*, accounting for 50.86% and 19.20% in 2015 (Table 1) as well as 46.3% and 17.3% in 2014 (Additional file 2: Table S1).

**Table 1:** Distribution of the IAI and UTI pathogens in China in 2015

	IAI			UTI			UTI + IAI		
	HA	CA	Total	HA	CA	Total	HA	CA	Total
*Enterobacteriaceae*	**928 (79.7)**	**287 (90.3)**	**1216 (82.0)** ^**a**^	**523 (87.3)**	**217 (92.7)**	**742 (88.9)** ^**a**^	**1451 (82.3)**	**504 (91.3)**	**1958 (84.5)** ^**a**^
*Escherichia coli*	477 (41.0)	170 (53.5)	648 (43.7)^a^	367 (61.3)	162 (69.2)	531 (63.6)^a^	844 (47.9)	332 (60.1)	1179 (50.9)^a^
*Klebsiella pneumoniae*	262 (22.5)	69 (21.7)	331 (22.3)	90 (15.0)	24 (10.3)	114 (13.7)	352 (20.0)	93 (16.9)	445 (19.2)
*Enterobacter cloacae*	83 (7.1)	17 (5.4)	100 (6.7)	12 (2.0)	4 (1.7)	16 (1.9)	95 (5.4)	21 (3.8)	116 (5.0)
*Proteus mirabilis*	21 (1.8)	8 (2.5)	29 (2.0)	15 (2.5)	11 (4.7)	26 (3.1)	36 (2.0)	19 (3.4)	55 (2.4)
*Citrobacter freundii*	16 (1.4)	3 (0.9)	19 (1.3)	12 (2.0)	6 (2.6)	18 (2.2)	28 (1.6)	9 (1.6)	37 (1.6)
*Enterobacter aerogenes*	24 (2.1)	3 (0.9)	27 (1.8)	8 (1.3)	1 (0.4)	9 (1.1)	32 (1.8)	4 (0.7)	36 (1.6)
Non-*Enterobacteriaceae*	**236 (20.3)**	**31 (9.8)**	**267 (18.0)**	**76 (12.7)**	**17 (7.3)**	**93 (11.2)**	**312 (17.7)**	**48 (8.7)**	**360 (15.5)**
*Pseudomonas aeruginosa*	104 (8.9)	14 (4.4)	118 (8.0)	44 (7.4)	9 (3.9)	53 (6.4)	148 (8.4)	23 (4.2)	171 (7.4)
*Acinetobacter baumannii*	103 (8.9)	12 (3.8)	115 (7.8)	20 (3.3)	5 (2.1)	25 (3.0)	123 (7.0)	17 (3.1)	140 (6.0)
*Stenotrophomonas maltophilia*	16 (1.4)	3 (0.9)	19 (1.3)	4 (0.7)	1 (0.4)	5 (0.6)	20 (1.1)	4 (0.7)	24 (1.0)
Other	58 (5.0)	19 (6.0)	77 (5.2)	27 (4.5)	11 (4.7)	38 (4.6)	85 (4.8)	30 (5.4)	115 (5.0)
All	**1164 (100)**	**318 (100)**	**1483 (100)**	**599 (100)**	**234 (100)**	**835 (100)**	**1763 (100)**	**552 (100)**	**2318 (100)**

^a^32 isolates lacked partial demographic information and could not be identified as CA or HA

**Fig. 2:**
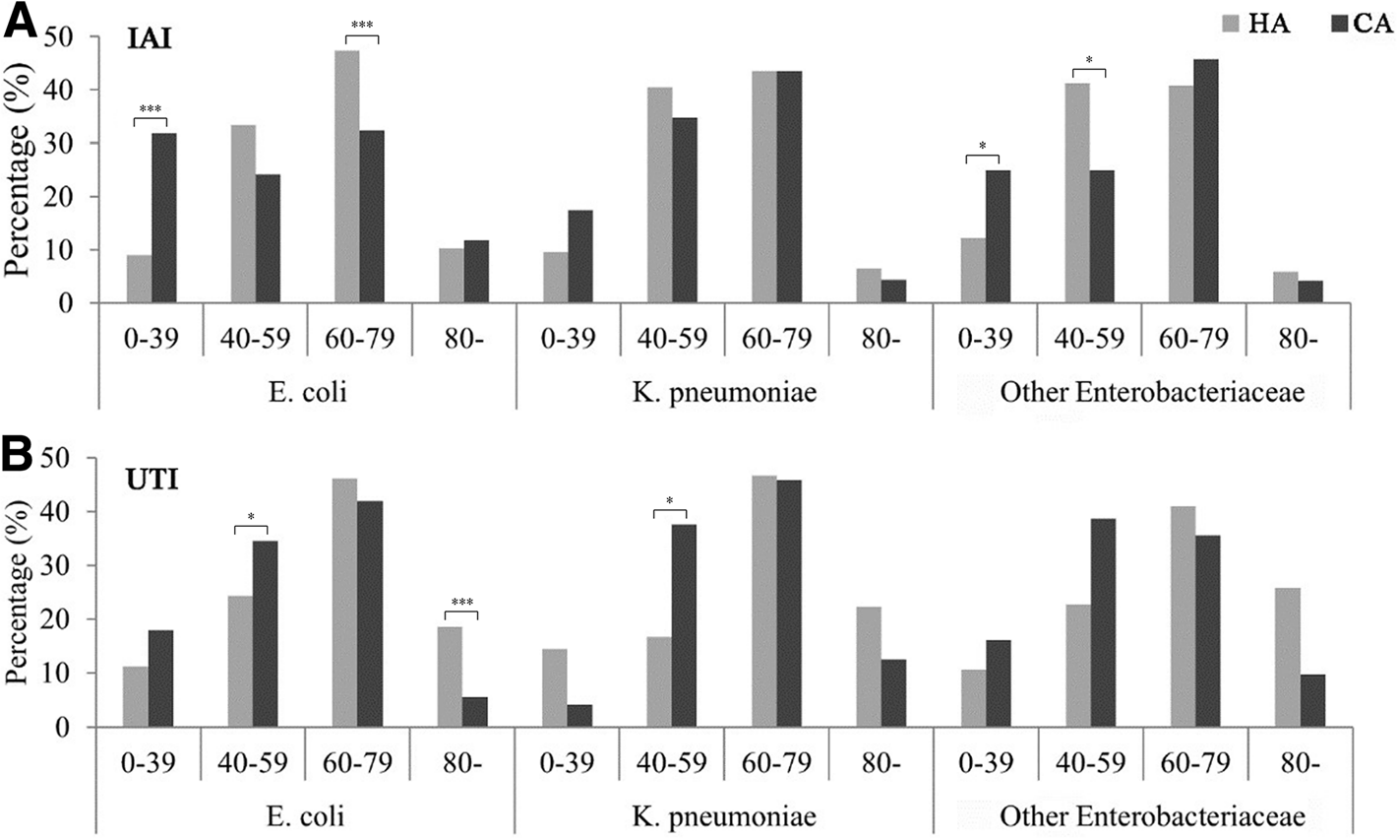
Distribution of *E. coli*, *K. pneumoniae* and other *Enterobacteriaceae* strains derived from HA and CA **a**) IAIs and **b**) UTIs in different age groups in 2015. * *P* < 0.05, *** *P* < 0.001

The distribution analysis of strains acquired from IAIs and UTIs in HA and CA in different age groups revealed that *E. coli* was the major IAI pathogen in CA IAI in the 0–39 year age range (CA: 31.76%; HA: 9.01%) and in HA IAI in the 60–79 years age range (CA: 32.35%; HA: 47.38%), as well as in the HA UTI of > 80 year-old patients (CA: 5.56%; HA: 18.53%) (Fig. 2), which was similar in 2014 (Additional file 3: Figure S2).

### Comparison of susceptibilities to 12 common antibiotics for *E. coli* and *K. pneumoniae* isolates (IAI and UTI) in 2014 and 2015

**Fig. 3:**
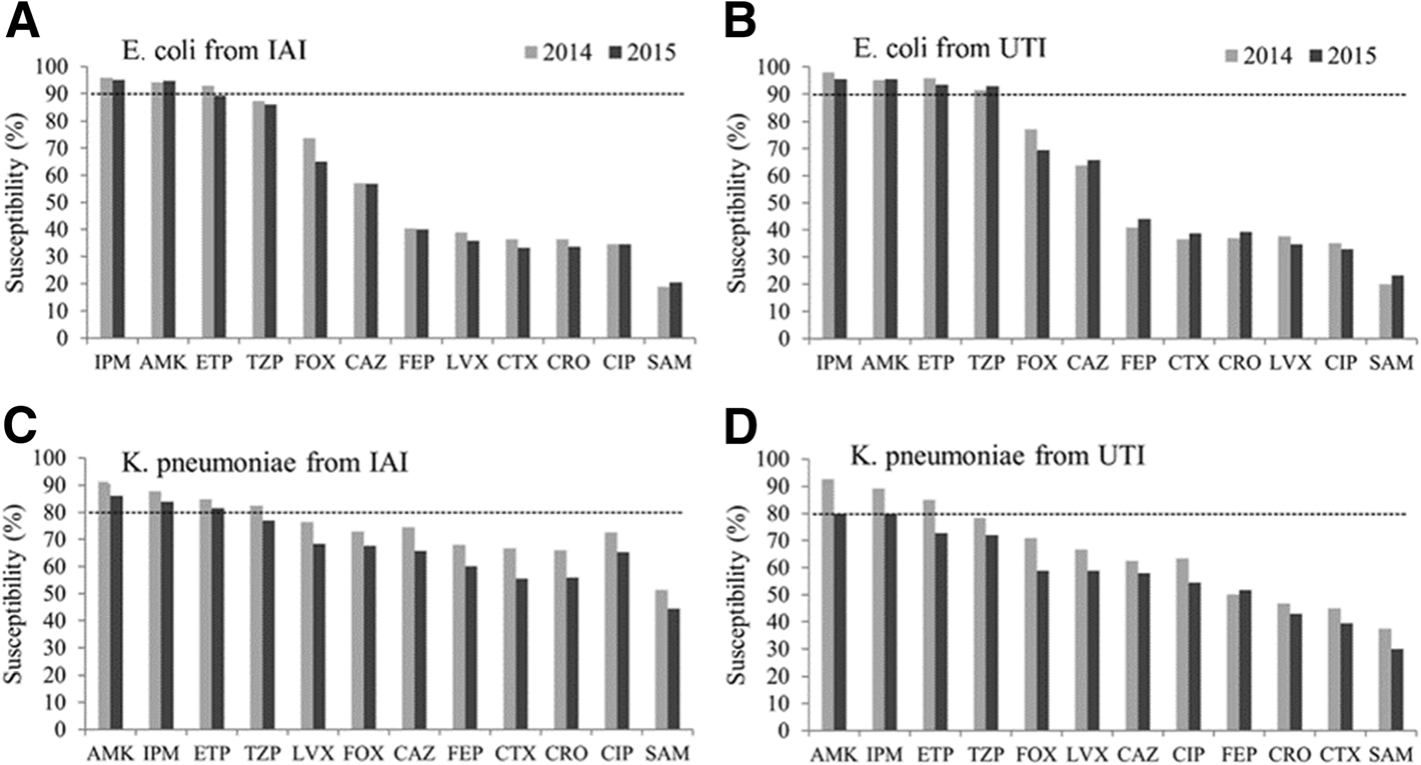
Comparison of *E. coli* and *K. pneumonia* isolate susceptibilities (IAI and UTI) to 12 common antibiotics between 2014 and 2015. **a** *E. coli* isolated from IAI. **b** *E. coli* isolated from UTI patients. **c** *K. pneumoniae* isolated from IAI patients. **d** *K. pneumoniae* isolated from UTI patients. Dotted lines show the indicated percentages throughout the columns for comparison

*E. coli* in IAI were highly susceptible to IPM, AMK and ETP (> 90%) in 2014, but the susceptibility to ETP showed a small decrease in 2015 (Fig. 3a) compared to 2014. *K. pneumoniae* from IAI was also highly sensitive to AMK, IPM and ETP (> 80%) in 2014–2015, but the susceptibility to these three antibiotics in 2015 was generally lower than in 2014 (Fig. 3c)*.* More than 90% *E. coli* isolates from UTI were susceptible to IPM, AMK, ETP and TZP. In contrast, compared to 2014 susceptibilities of *K. pneumoniae* strains from UTIs decreased by 10% to AMK, IPM and ETP in 2015, which was a general trend also for all the other antibiotics tested (Fig. 3d). Susceptibilities to all other antibiotics were between 20 and 70% for *E. coli* and 30–75% for *K. pneumoniae* (Fig. 3). Corresponding MIC_90_ values of 12 antibiotics for *E. coli* and *K. pneumoniae* IAI and UTI isolates are presented in the Additional file 4: Table S2.

### Comparison of the Enterobacteriaceae susceptibilities to carbapenem antibiotics (ETP and IPM) based on the OSWIA in different organs of IAI patients as well as isolates derived from IAI and UTI patients in different departments between 2014 and 2015

**Fig. 4:**
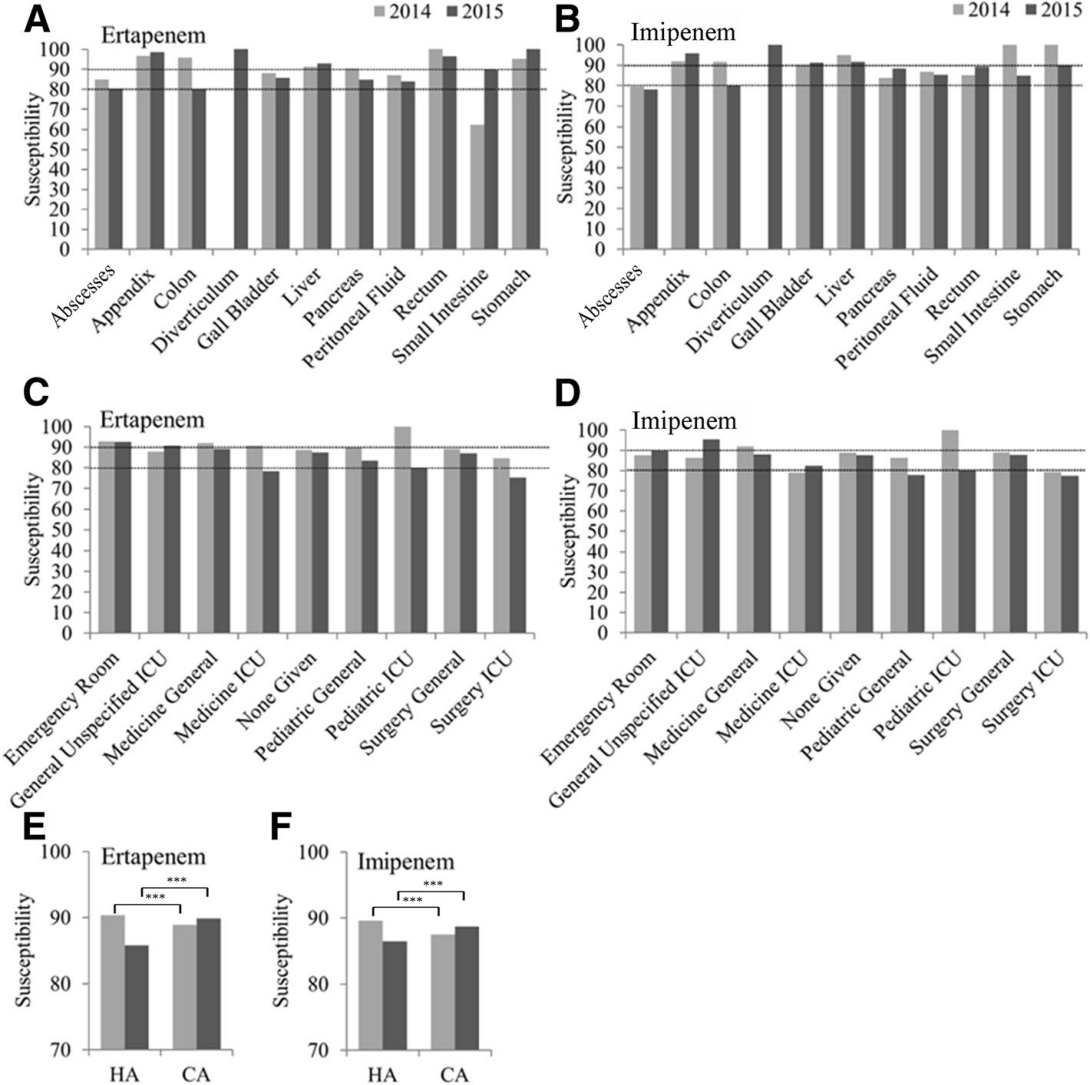
Susceptibility based on OSWIA in IAI isolates. **a** OSWIA susceptibility of *Enterobacteriaceae* IAI to ETP. **b** OSWIA susceptibility of *Enterobacteriaceae* IAI to IPM. **c** Susceptibilities of *Enterobacteriaceae* UTI and IAI strains to ETP. **d** to IPM. **e** *Enterobacteriaceae* susceptibilities of HA and CA IAI and UTI to ETP. **f** *Enterobacteriaceae* susceptibilities of HA and CA IAI and UTI to IPM. *** *P* < 0.001

In general, besides abscesses and colon infections in 2015 as well as small intestine infections in 2014 to ETP, most included *Enterobacteriaceae* IAI isolates showed OSWIA susceptibilities to carbapenems of > 80% in 2014 and 2015 (Fig. 4a**,** Fig. 4b).

In 2015, carbapenem susceptibility rates of *Enterobacteriaceae* isolated from IAIs and UTIs decreased below 80% in medical and surgery ICUs to ETP as well as in general pediatric departments and surgery ICUs in 2015 to IPM. The greatest decline in susceptibility to IPM and ETP was seen between 2014 and 2015 in pediatric ICUs (Fig. 4c**,** Fig. 4d). In general there was a trend of susceptibility reductions to ETP and IPM in HA, and susceptibility increases to ETP and IPM in CA-derived *Enterobacteriaceae* caused IAIs between 2014 and 2015 (Fig. 4e**,** Fig. 4f).

Detailed information of MIC_90_ values from *E. coli* and *K. pneumoniae* for ETP and IMP in different organs, departments and HA vs CA infections in 2014 and 2015 are shown in Table 2 and indicate dramatically increased MIC_90_ values of IPM for *E. coli* isolates only in medicine ICUs in 2015, while the MIC_90_ values for IPM of *K. pneumoniae* IAI isolates from abscesses, colon, peritoneal fluid and others were all > 32 in 2015, pointing out increasing IPM resistance of *K. pneumoniae* infections in 2015. In addition, the MIC_90_ values of IPM for *K. pneumoniae* isolates derived from general surgery departments as well as from ICUs of medicine, pediatric and surgery became > 32 and also reflected in essentially increased HA MIC_90_ values (Table 2), which showed that clinical relevant resistance of IAI derived *K. pneumoniae* isolates to IPM appeared in hospitals in 2015.

**Table 2:** MIC_90_ values of ETP and IMP for *E. coli* and *K. pneumoniae* in 2014 and 2015

		ETP		IPM	
		2014	2015	2014	2015
*E. coli*	Abscesses	0.5	2	0.5	1
	Appendix	0.12	0.25	0.25	1
	Colon	0.5	0.25	0.25	≤ 0.5
	Gall Bladder	0.5	1	0.25	1
	Liver	2	0.5	0.5	≤ 0.5
	Pancreas	1	0.5	0.12	≤ 0.5
	Peritoneal Fluid	0.5	0.5	0.5	1
	Rectum	0.25	0.5	2	1
	Small Intestine	1	0.5	0.25	≤ 0.5
	Stomach	≤ 0.03	0.06	0.25	≤ 0.5
	Other	0.5	> 4	0.5	> 32
	Emergency Room	0.12	0.5	0.25	1
	General Unspecified ICU	0.5	1	1	1
	Medicine General	0.5	1	0.25	1
	Medicine ICU	≤ 0.03	4	0.25	> 32
	None Given	0.06	1	0.12	≤ 0.5
	Pediatric General	0.12	≤ 0.03	1	1
	Pediatric ICU	≤ 0.03	≤ 0.03	0.12	1
	Surgery General	0.5	0.5	0.5	1
	Surgery ICU	0.25	2	0.25	1
	HA	0.5	1	0.5	1
	CA	0.5	0.5	0.5	1
*K. pneumoniae*	Abscesses	> 4	> 4	> 8	> 32
	Appendix	1	0.06	0.5	1
	Colon	≤ 0.03	> 4	0.25	> 32
	Diverticulum		≤ 0.03		≤ 0.5
	Gall Bladder	2	> 4	2	8
	Liver	0.06	≤ 0.03	0.5	1
	Pancreas	0.25	0.25	8	1
	Peritoneal Fluid	> 4	> 4	> 8	> 32
	Rectum	0.12	0.12	0.12	2
	Small Intestine	4		0.12	≤ 0.5
	Stomach	0.25		0.5	1
	Other	> 4	> 4	> 8	> 32
	Emergency Room	1	0.5	2	2
	General Unspecified ICU	> 4	0.25	> 8	1
	Medicine General	0.5	4	1	2
	Medicine ICU	> 4	> 4	> 8	> 32
	None Given	≤ 0.03	0.06	0.12	≤ 0.5
	Pediatric General	≤ 0.03	≤ 0.03	0.25	≤ 0.5
	Pediatric ICU		> 4		> 32
	Surgery General	> 4	> 4	8	32
	Surgery ICU	> 4	> 4	> 8	> 32
	HA	1	> 4	1	> 32
	CA	> 4	4	> 8	2

## Discussion

*Enterobacteriaceae* were the major pathogens in IAI and UTI, with *E. coli* and *K. pneumoniae* being the most commonly isolated strains, which is in accordance with recent studies in China and abroad [3, 9] [10]. *E. coli* isolated from both UTI and IAI were < 40% susceptible to the tested fluoroquinolones that reflects an overuse of fluoroquinolones in China, which has also been reported for Europe and the US [11, 12]. In addition, in 2015 *E. coli* isolates were < 70% susceptible to all cephalosporins tested including cefoxitin whether they were obtained from IAI or UTI, suggesting a high prevalence of ESBL production, which is an extension of the trend shown previously between 2002 and 2011 [1]. A similar but less dramatic pattern has been observed for *K. pneumoniae*, but in contrast to *E. coli*, *K. pneumoniae* showed a decreasing susceptibility to all tested antibiotics from 2014 to 2015 (Fig. 3). Though ESBL-producing *E. coli* and *K. pneumoniae* strains should be susceptible to cefoxitin, both species, whether found in IAI or UTI, were < 80% susceptible to cefoxitin, which suggests that besides ESBL production other resistance mechanisms may be on the rise [13]. In particular *K. pneumoniae* showed a decreasing susceptibility to carbapenems, which was more pronounced in UTI isolates and indicated that carbapenemases or other mechanisms of carbapenem resistance have developed in *K. pneumoniae* strains, which has also been previously noted since carbapenem resistant *K. pneumoniae* strains isolated in Shanghai between July 2014 and May 2015 harbored all or at least one of the ESBL genes plus mainly New Delhi metallo-β-lactamase-1 (NDM-1) and IMP-4 or *Klebsiella pneumoniae* carbapenemase (KPC)-2 [14].

However, data from 2015 showed *K. pneumoniae* IAI isolate susceptibilities between 80 and 90% to IPM and ETP in our study, which was much higher than the 43.24% susceptibility to IPM reported for *K. pneumoniae* IAI isolates from a Chinese tertiary-care hospital between 2012 and 2015 [15], but was in a similar range to Chinese isolates from abdominal trauma-associated IAI collected between 2010 to 2015, with susceptibilities of *K. pneumoniae* strains to IPM and ETP of 87.5–90.6% [16].

The distribution of *Enterobacteriaceae* IAI isolates were similar in peritoneal fluid, abscesses and the colon for HA and CA infections, but HA IAIs manifested more in the gall bladder whereas CA infections occurred to a greater extent in appendicitis IAIs in 2015 (Fig. 1), which is a fair finding since acute appendicitis is a common reason for hospital admissions worldwide [17]. In particular, colon IAI-derived strains showed an increased resistance to carbapenems, decreasing to 80% susceptibility to ertapenem and imipenem in 2015. Similarly, IAI strain susceptibilities from abscesses were about 80% to carbapenems in 2015, but the decrease compared to 2014 was less pronounced (Fig. 3). Apart from general pediatric departments, in 2015 lowered susceptibilities particularly to IPM were most obvious in ICUs, which is in agreement with previous studies, in which it was noted that patients infected with carbapenem-resistant *K. pneumoniae* strains were mainly elderly, possessed multiple co-morbidities, were frequently admitted from and discharged to post-acute care facilities, and experienced prolonged hospital stays [18]. In addition, for carbapenem-resistant Gram-negative pathogen infections, previous administration of carbapenems has been shown to be a major factor, particularly in ICUs [19], and isolation of patients harboring carbapenem-resistant *Enterobacteriaceae* and delayed application of alternative antibiotics has been proposed to lead to a spread of these pathogens in ICUs [20].

A limitation of the present study is the missing data about molecular mechanisms of resistances against the included antibiotics.

## Conclusions

Susceptibilities of *E. coli* IAI and UTI strains to IPM and ETP were > 90% in 2014 and 2015, while susceptibilities to IPM and ETP of *K. pneumoniae* IAI strains were >  80% in 2014, but decreased to ≤80% in 2015, particularly for UTI strains. Susceptibilities of all IAI *Enterobacteriaceae* strains to IPM and ETP were lowest in the colon and abscesses and *Enterobacteriaceae* susceptibilities of both UTI and IAI isolates to IPM and ETP were lowest in medical, pediatric and surgery ICUs in 2015.

Susceptibilities rates of *E. coli* and *K. pneumoniae* strains to cephalosporins collected from UTIs ranged from 38.6 to 69.5% and for IAI strains from 33.18 to 67.7% in 2015, which suggests that cephalosporins should not be the first choice for empirical UTI and IAI antibiotic therapy.

## Additional files

Additional file 1: **Figure S1.** Distribution of isolates acquired from IAI pathogens in different organ groups in 2014. (TIF 357 kb)

Additional file 2: **Table S1.** Distribution of the IAI and UTI pathogens in China in 2014. (DOCX 18 kb)

Additional file 3: **Figure S2.** Distribution of *E. coli*, *K. pneumoniae* and other *Enterobacteriaceae* strains in different age groups in 2014. * *P* < 0.05, *** *P* < 0.001. (TIF 268 kb)

Additional file 4: **Table S2.** MIC_90_ values of *E.coli* and *K. pneumoniae* for the indicated antibiotics in 2014 and 2015. (DOCX 15 kb)

## Abbreviations

AMK: amikacin
CA: community-associated
CAZ: ceftazidime
CIP: ciprofloxacin
CIs: confidence intervals
CLSI: Clinical and Laboratory Standards Institute
CRO: ceftriaxone
CTX: cefotaxime
*E. coli*: *Escherichia coli*
ETP: ertapenem
FEP: cefepime
FOX: cefoxitin
IAI: intra-abdominal infections
IPM: imipenem
*K. pneumoniae*: *Klebsiella pneumoniae*
LVX: levofloxacin
MIC: Minimum inhibitory concentrations
OSWIA: organ-specific weighted incidence antibiograms
QC: quality control
SAM: ampicillin-sulbactam
SMART: Study for Monitoring Antimicrobial Resistance Trends
TZP: piperacillin-tazobactam
UTI: urinary tract infections
