# Supplementary figures and images for: Carbapenem susceptibilities of Gram-negative pathogens in intra-abdominal and urinary tract infections: updated report of SMART 2015 in China
Source: BMC Infect Dis. 2018 Sep 29;18:493. doi: 10.1186/s12879-018-3405-1 (PMC6162895; doi:10.1186/s12879-018-3405-1)

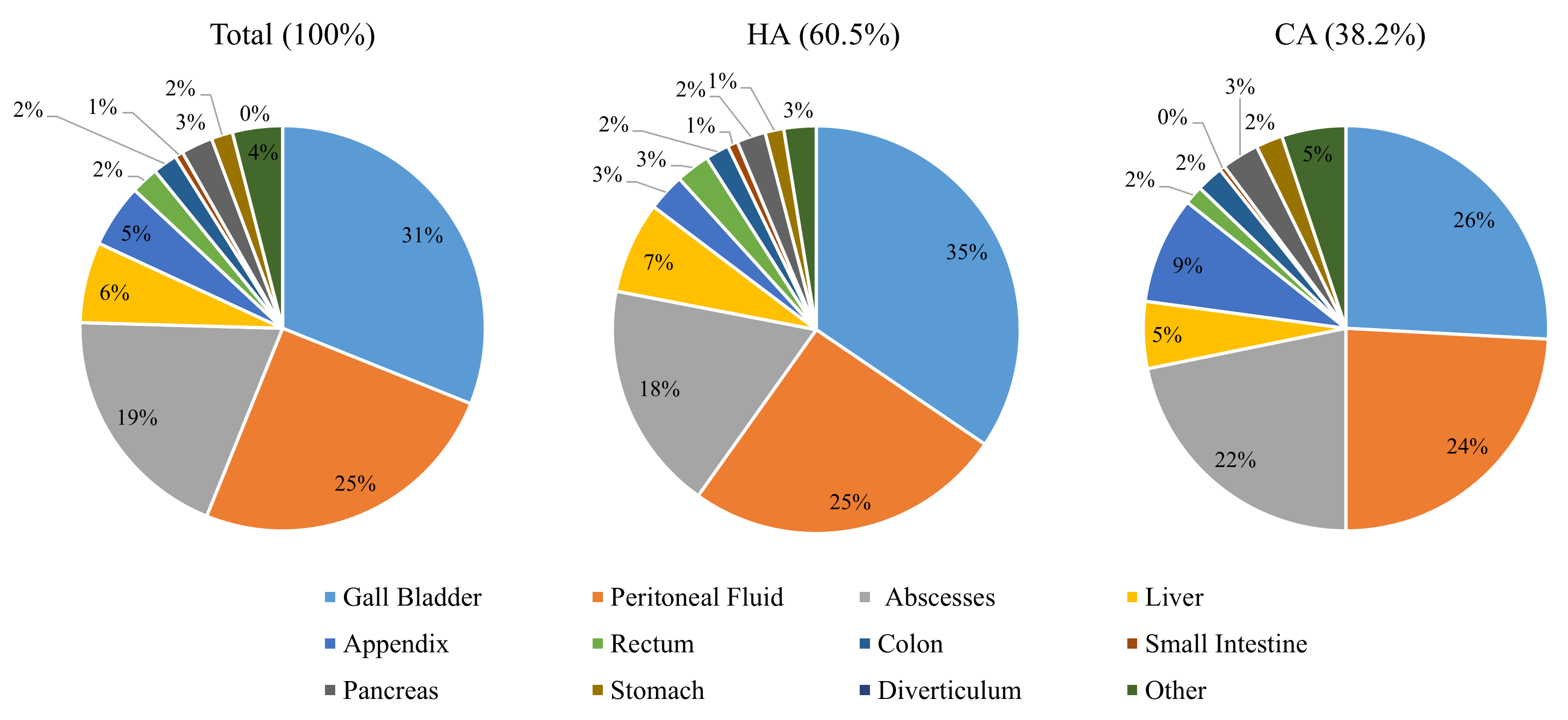

Supplement: Supplementary file 1 — Figure S1. Distribution of isolates acquired from IAI pathogens in different organ groups in 2014. (TIF 357 kb) [file 12879_2018_3405_MOESM1_ESM.tif]

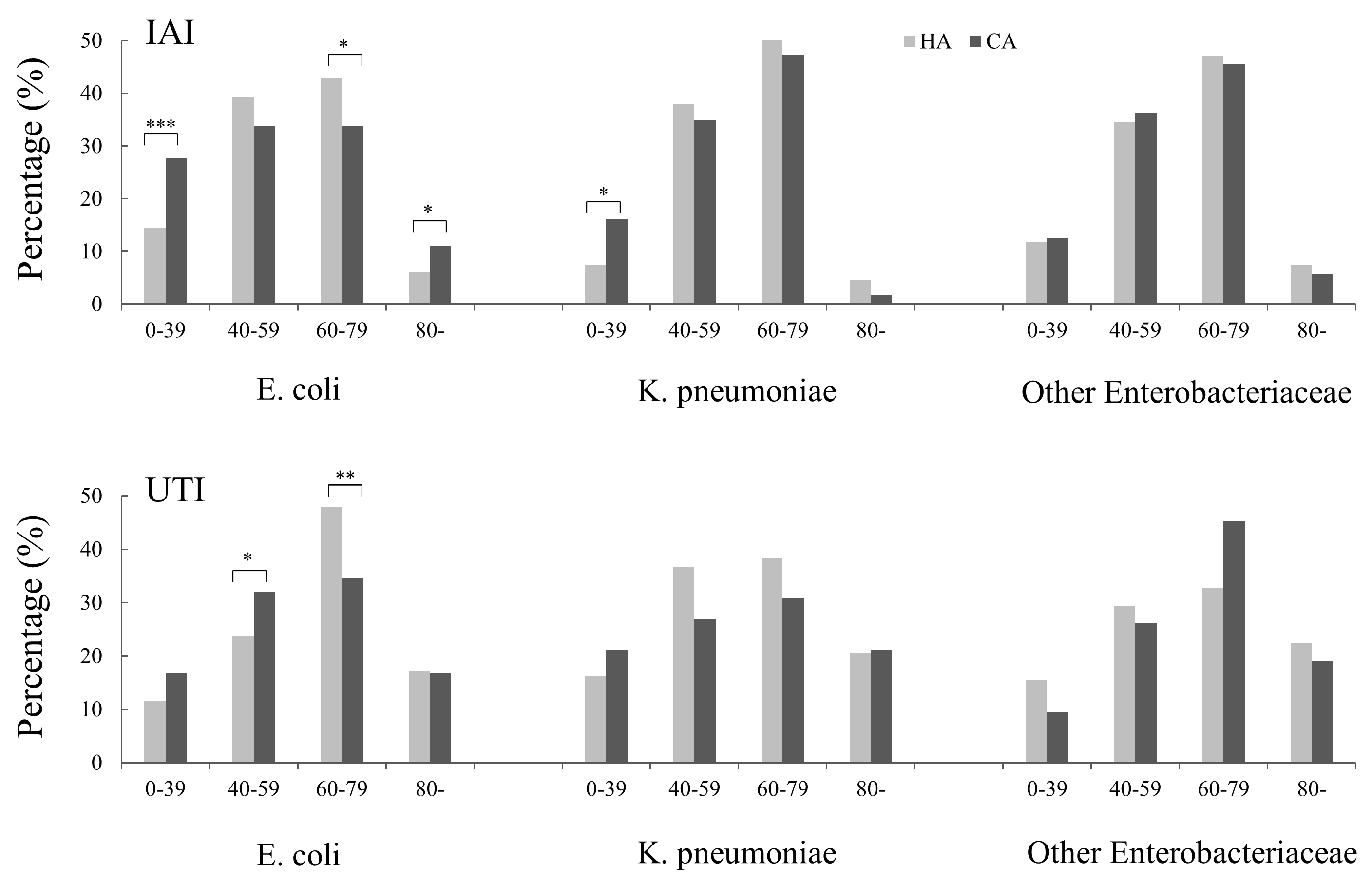

Supplement: Supplementary file 3 — Figure S2. Distribution of E. coli, K. pneumoniae and other Enterobacteriaceae strains in different age groups in 2014. * P < 0.05, *** P < 0.001. (TIF 268 kb) [file 12879_2018_3405_MOESM3_ESM.tif]
