# Supplementary material for: Carbapenem susceptibilities of Gram-negative pathogens in intra-abdominal and urinary tract infections: updated report of SMART 2015 in China
Source: BMC Infect Dis. 2018 Sep 29;18:493. doi: 10.1186/s12879-018-3405-1 (PMC6162895; doi:10.1186/s12879-018-3405-1)
Supplement: Supplementary file 2 — Table S1. Distribution of the IAI and UTI pathogens in China in 2014. (DOCX 18 kb) [file 12879_2018_3405_MOESM2_ESM.docx]

**Table 1**. Distribution of the IAI and UTI pathogens in China in 2014

|  | IAI | | | UTI | | | UTI+IAI | | |
| --- | --- | --- | --- | --- | --- | --- | --- | --- | --- |
|  | HA | CA | **Total** | HA | CA | **Total** | HA | CA | **Total** |
| **Enterobacteriaceae** | **795 (80.3)** | **523 (82.0)** | **1345 (81.1)*** | **440 (87.1)** | **315 (88.2)** | **762 (87.6)*** | **1235 (82.7)** | **838 (84.2)** | **2107 (83.3)*** |
| *Escherichia coli* | 405 (41.0) | 268 (42.0) | 682 (41.1)* | 287 (56.8) | 197 (55.2) | 489 (56.2)* | 692 (46.3) | 465 (46.7) | 1171 (46.3)* |
| *Klebsiella pneumoniae* | 200 (20.2) | 112 (17.6) | 318 (19.2)* | 68 (13.5) | 52 (14.6) | 120 (13.8) | 268 (17.9) | 164 (16.5) | 438 (17.3)* |
| *Enterobacter cloacae* | 57 (5.8) | 23 (3.6) | 80 (4.8) | 13 (2.6) | 10 (2.8) | 23 (2.6) | 70 (4.7) | 33 (3.3) | 103 (4.1) |
| *Proteus mirabilis* | 20 (2.0) | 9 (1.4) | 29 (1.7) | 14 (2.8) | 8 (2.2) | 23 (2.6)* | 34 (2.3) | 17 (1.7) | 52 (2.1)* |
| *Citrobacter freundii* | 17 (1.7) | 14 (2.2) | 32 (1.9)* | 7 (1.4) | 6 (1.7) | 13 (1.5) | 24 (1.6) | 20 (2.0) | 45 (1.8)* |
| *Klebsiella aerogenes* | 17 (1.7) | 14 (2.2) | 31 (1.9) | 6 (1.2) | 3 (0.8) | 10 (1.1)* | 23 (1.5) | 17 (1.7) | 41 (1.6)* |
| *Klebsiella oxytoca* | 12 (1.2) | 18 (2.8) | 30 (1.8) | 13 (2.6) | 10 (2.8) | 23 (2.6) | 25 (1.7) | 28 (2.8) | 53 (2.1) |
| *Morganella morganii* | 14 (1.4) | 10 (1.6) | 24 (1.4) | 5 (1.0) | 5 (1.4) | 10 (1.1) | 19 (1.3) | 15 (1.5) | 34 (1.3) |
| **Non-Enterobacteriaceae** | **194 (19.6)** | **115 (18.0)** | **314 (18.9)*** | **65 (12.9)** | **42 (11.8)** | **108 (12.4)*** | **259 (17.3)** | **157 (15.8)** | **422 (16.7)*** |
| *Pseudomonas aeruginosa* | 94 (9.5) | 61 (9.6) | 157 (9.5)* | 33 (6.5) | 25 (7.0) | 58 (6.7) | 127 (8.5) | 86 (8.6) | 215 (8.5)* |
| *Acinetobacter baumannii* | 70 (7.1) | 39 (6.1) | 112 (6.8)* | 19 (3.8) | 10 (2.8) | 29 (3.3) | 89 (6.0) | 49 (4.9) | 141 (5.6)* |
| *Stenotrophomonas maltophilia* | 10 (1.0) | 6 (0.9) | 16 (1.0) | 2 (0.4) | 1 (0.3) | 3 (0.3) | 12 (0.8) | 7 (0.7) | 19 (0.8) |
| others | 84 (8.5) | 64 (10.0) | 148 (8.9) | 38 (7.5) | 30 (8.4) | 69 (7.9)* | 122 (8.2) | 94 (9.4) | 217 (8.6)* |
| **All** | **989 (100)** | **638 (100)** | **1659 (100)** | **505 (100)** | **357 (100)** | **870 (100)** | **1494 (100)** | **995 (100)** | **2529 (100)** |

*40 isolates lacked partial demographic information and could not be identified as CA or HA.
