# Supplementary material for: Carbapenem susceptibilities of Gram-negative pathogens in intra-abdominal and urinary tract infections: updated report of SMART 2015 in China
Source: BMC Infect Dis. 2018 Sep 29;18:493. doi: 10.1186/s12879-018-3405-1 (PMC6162895; doi:10.1186/s12879-018-3405-1)
Supplement: Supplementary file 4 — Table S2. MIC90 values of E.coli and K. pneumoniae for the indicated antibiotics in 2014 and 2015. (DOCX 15 kb) [file 12879_2018_3405_MOESM4_ESM.docx]

Supplementary Table 2. MIC_90_ values of *E.coli* and *K. pneumoniae* for the indicated antibiotics in 2014 and 2015

|  |  | *E.coli* | | | *K.pneumoniae* | |
| --- | --- | --- | --- | --- | --- | --- |
|  |  | 2014 | 2015 | 2014 | | 2015 |
| IAI | AMK | 8 | 8 | 8 | | > 32 |
|  | SAM | > 16 | > 16 | > 16 | | > 16 |
|  | FEP | > 32 | > 32 | > 32 | | > 32 |
|  | CTX | > 128 | > 32 | > 128 | | > 32 |
|  | FOX | > 16 | > 16 | > 16 | | > 16 |
|  | CAZ | > 128 | > 32 | 128 | | > 32 |
|  | CRO | > 32 | > 32 | > 32 | | > 32 |
|  | CIP | > 2 | > 2 | > 2 | | > 2 |
|  | ETP | 0.5 | 1 | > 4 | | > 4 |
|  | IPM | 0.5 | 1 | 8 | | > 32 |
|  | LVX | > 4 | > 4 | > 4 | | > 4 |
|  | TZP | 64 | 64 | > 64 | | > 64 |
| UTI | AMK | 8 | 8 | 8 | | > 32 |
|  | SAM | > 16 | > 16 | > 16 | | > 16 |
|  | FEP | > 32 | > 32 | > 32 | | > 32 |
|  | CTX | > 128 | > 32 | > 128 | | > 32 |
|  | FOX | > 16 | > 16 | > 16 | | > 16 |
|  | CAZ | 128 | 32 | 128 | | > 32 |
|  | CRO | > 32 | > 32 | > 32 | | > 32 |
|  | CIP | > 2 | > 2 | > 2 | | > 2 |
|  | ETP | 0.25 | 0.25 | 2 | | > 4 |
|  | IPM | 0.25 | 1 | 2 | | > 32 |
|  | LVX | > 4 | > 4 | > 4 | | > 4 |
|  | TZP | 16 | 16 | > 64 | | > 64 |
